# An unusual case of intraorbital foreign body and its management

**DOI:** 10.4103/0301-4738.73725

**Published:** 2011

**Authors:** Bipasha Mukherjee, Shubhra Goel, Nirmala Subramanian

**Affiliations:** Medical Research Foundation, Sankara Nethralaya, Chennai, India

**Keywords:** Orbital foreign body, plastic, thermal injury

## Abstract

Intraorbital foreign bodies are usually the result of accidental trauma and can lead to considerable morbidity. We report an unusual case of an industrial injury in a plastic manufacuring unit wherein hot molten plastic splashed and solidified inside the orbit. The resultant increased intraorbital pressure led to loss of vision in that eye. The extreme temperature of the foreign body caused extensive thermal damage to the surrounding adnexal structures. Staged reconstructive surgery was undertaken to repair the damage, with an acceptable final cosmetic outcome. Employment of protective eye wear to prevent such accidents in high-risk occupations should be made mandatory.

Judicious use of protective eye glasses can prevent blindness in workplace-related trauma. We report a unique case of plastic intraorbital foreign body (IOrFB). Widespread immediate and delayed damage was caused by the sheer size and temperature of the molten plastic that solidified inside the orbit. Although staged reconstruction ultimately resulted in satisfactory cosmesis, the patient lost vision in that eye. Plastic IOrFBs are rarely reported in the literature.[1] A case such as this of a large plastic IOrFB causing blindness is hitherto unreported.

## Case Report

A 25-year-old male who suffered a work-related accident in a plastic manufacturing industry was brought to our emergency department after hot molten plastic splashed into his right eye. He was brought 6 h after the incident. The patient complained of immediate vision loss associated with severe pain and a burning sensation. According to the patient, he was not using any protective glasses and the temperature of the molten polymer was 240 degrees Fahrenheit. No first-aid was administered to him during this period.

**Figure 1:**
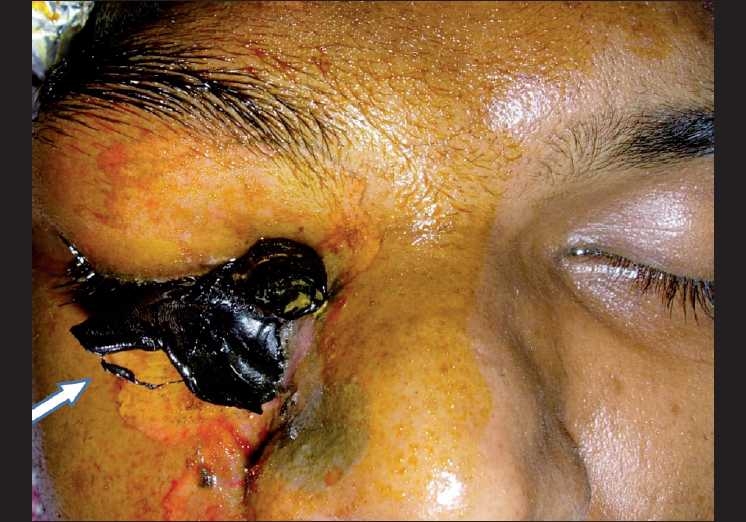
Solidified polymer protruding from the skin of the right lower eyelid

On examination, he had no light perception in his right eye. The left eye vision was 20/20; J1. The right eye showed severe periocular edema with a black hardened plastic foreign body protruding from the lower lid and medial canthal area [Fig. 1]. Superficial burns involving the right cheek were evident. Extraocular movements were restricted in all gazes Examination of the anterior segment of the right eye showed conjunctival congestion and chemosis with diffuse corneal edema. The pupil was fixed and mid-dilated with normal anterior chamber depth. Intraocular pressure (IOP) with tonopen was 86 mmHg. An examination of the fundus showed hyperemic optic disc with blurred margins, diffuse retinal edema with scattered pre- and sub-retinal hemorrhages.

**Figure 2:**
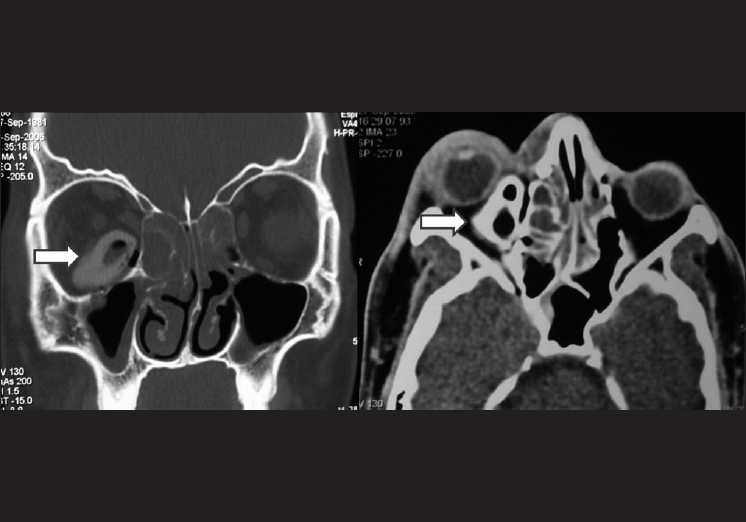
Computed tomography scan showing the intraorbital plastic foreign body in the right orbit with the air pockets (arrows)

The patient was started on actazolamide 500 mg tablets and timolol maleate 0.5% eye drops. Lateral canthotomy with cantholysis of both crura of lateral canthal tendon was performed as well. A computed tomography (CT) scan was ordered, which showed a hyper dense IOrFB in the inferomedial quadrant of the right orbit, displacing the globe upwards. Hypodense areas inside the mass were evident, suggestive of air entrapment [Fig. 2]. There was no extension to the sinuses or the nasal cavity, and the globe was intact. The optic nerve was not clearly visualized.

**Figure 3:**
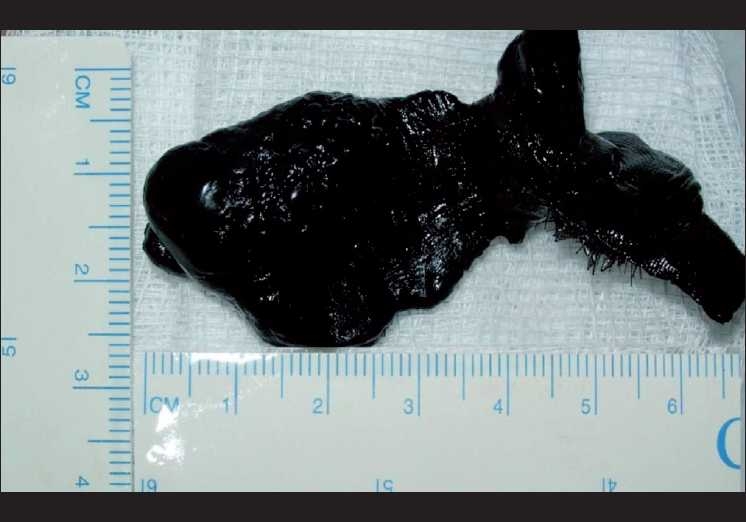
Plastic foreign body after removal

The patient was operated under general anesthesia. The hardened plastic material was lying embedded at the right medial canthal area and the lower lid. The entry wound was enlarged and the foreign body was removed *in toto*. It measured 2.5 × 3.5 × 5.0 cm [Fig. 3]. The globe was found to be intact. However, the wound depth extended down to the periosteum of the inferior orbital rim with extensive edema of the surrounding skin. The lower lid wound was sutured in one layer using interrupted 6-0 nylon sutures.

Postoperatively, the patient was administered intravenous injections of cefotaxime (1 gm) and dexamethasone (4 mg) twice daily in the ward. On the second postoperative day, the wound was healthy. The cornea was clear and IOP was 18 mmHg. The pupil remained dilated and fixed. B-scan ultrasonography (USG) showed widening of Tenon’s space, suspicious of blood clot around the optic nerve. Three daily doses of intravenous methyl prednisolone (1 gm/day) were administered, but the patient reported no improvement in his vision. The patient was discharged on Ciprofloxacin tablets 1 gm/day and prednisolone tablets (1 gm/kg body weight) in a weekly tapering dose.

**Figure 4:**
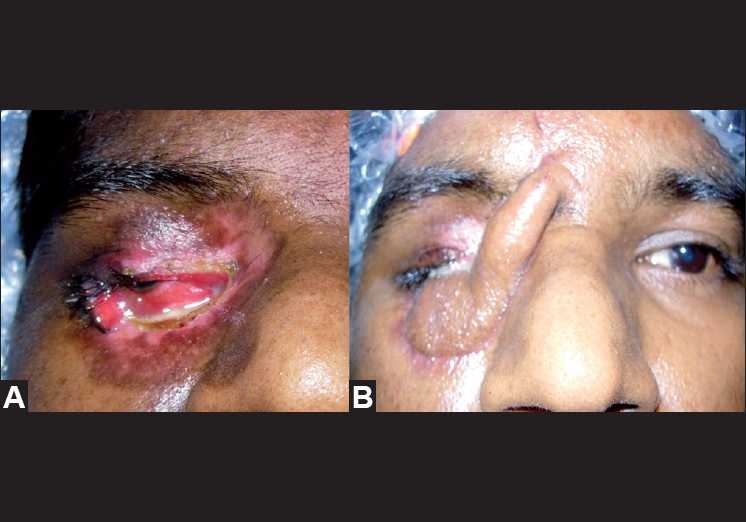
(A) Loss of skin over the right lower lid, medial upper lid and medial canthus. (B) Reconstruction by tarsoconjunctival and medial forehead flap

**Figure 5:**
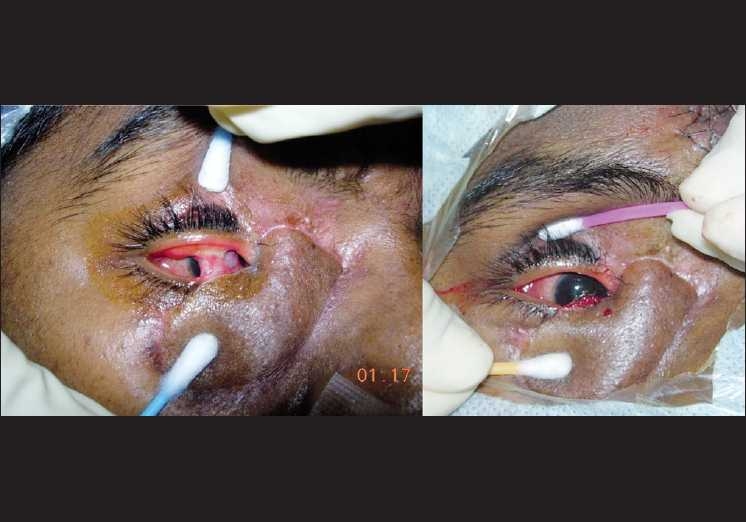
Tarsoconjunctival flap after division

**Figure 6:**
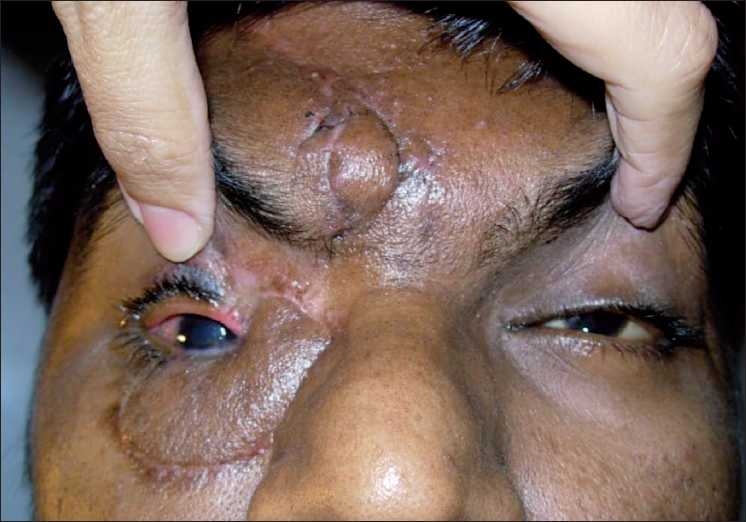
Final result

At the 3-week follow-up consultation, the skin of the medial three-fourth of the lower lid surrounding the entry wound had sloughed out. The necrosis of the tissues extended up to the orbital rim, medial portion of the upper lid and the medial canthal area [Fig. 4A]. Wound debridement was required and broad-spectrum systemic antibiotics (capsule ampicillin + dicloxacillin 500 mg twice/day) was restarted. Fundus examination showed resolution of retinal hemorrhages and optic atrophy. A staged reconstruction of the right medial canthal area, including the upper and lower eyelids, was performed using a combination of forehead and tarso-conjunctival flaps techniques [Figs. 4B and 5]. Although acceptable cosmesis was achieved [Fig. 6], the patient did not recover his vision. Protective polycarbonate glasses for constant wear was prescribed to the patient.

## Discussion

The clinical features and the management of IOrFBs vary, depending on their size, location, velocity and composition. Unlike intraocular foreign bodies, sterile inorganic IOrFBs may be left *in situ* safely.[2] However, they can cause acute vision loss due to the associated intraocular trauma (globe rupture) or traumatic optic neuropathy or late complications, such as orbital cellulitis (especially if organic), inflammation (copper), systemic toxicity (lead), siderosis (iron) and discharging sinus (wooden).[3]

Plastic, *per se*, is an inert polymer, but it caused devastating morbidity in our patient by virtue of its size and the temperature at the time of contact. The force of impact and the high temperature caused not only raised the IOP and, thus, intra-orbital pressure, leading to optic atrophy and also extensive thermal burns to the adnexal structures.

Severe thermal injuries of the eyes destroy the surface epithelia and cause ischemic necrosis and, later, contracture due to fibrosis.[4] In this case, the temperature of the foreign body at the time of entry was around 240 degrees Fahrenheit, causing third-degree thermal burns, resulting in sloughing and necrosis.

As the consequential tissue loss is not evident at presentation, these patients need to be counselled for the possible need for multiple surgeries later for cosmetic rehabilitation.

Reconstruction of the eyelids in our patient was started once the depth of the tissue damage due to burns became well demarcated.[5] The reconstructive surgery was undertaken in stages and the final result was cosmetically acceptable.

CT scan was the most useful imaging modality for localizing the IOrFB and delineating its posterior extent.

It also provided clues regarding the status of the globe and the optic nerve.

In conclusion, IOrFBs can cause significant ocular morbidity. Spreading awareness for the employment of protective measures in high-risk occupations should be made compulsory and would be helpful in reducing these injuries.[6]
